# Analysing Local Sparseness in the Macaque Brain Network

**DOI:** 10.1371/journal.pone.0138148

**Published:** 2015-10-05

**Authors:** Raghavendra Singh, Seema Nagar, Amit A. Nanavati

**Affiliations:** 1 IBM Research, India, New Delhi, Delhi, India; 2 IBM Research, India, Bangalore, Karnataka, India; Universidad Rey Juan Carlos, SPAIN

## Abstract

Understanding the network structure of long distance pathways in the brain is a necessary step towards developing an insight into the brain’s function, organization and evolution. Dense global subnetworks of these pathways have often been studied, primarily due to their functional implications. Instead we study sparse local subnetworks of the pathways to establish the role of a brain area in enabling shortest path communication between its non-adjacent topological neighbours. We propose a novel metric to measure the topological communication load on a vertex due to its immediate neighbourhood, and show that in terms of distribution of this local communication load, a network of Macaque long distance pathways is substantially different from other real world networks and random graph models. Macaque network contains the entire range of local subnetworks, from star-like networks to clique-like networks, while other networks tend to contain a relatively small range of subnetworks. Further, sparse local subnetworks in the Macaque network are not only found across topographical super-areas, e.g., lobes, but also within a super-area, arguing that there is conservation of even relatively short-distance pathways. To establish the communication role of a vertex we borrow the concept of brokerage from social science, and present the different types of brokerage roles that brain areas play, highlighting that not only the thalamus, but also cingulate gyrus and insula often act as “relays” for areas in the neocortex. These and other analysis of communication load and roles of the sparse subnetworks of the Macaque brain provide new insights into the organisation of its pathways.

## Introduction

White matter pathways in the brain mediate information flow and facilitate information integration and cooperation across functionally differentiated distributed centres of sensation, perception, action, cognition, and emotion. Extensive research on mapping the pathways, and analyzing the resultant network is presenting a new picture of the primate brain that views cognitive processes to be a result of collective and coordinated phenomena mediated by the pathways [1, 2].

**Fig 1:**
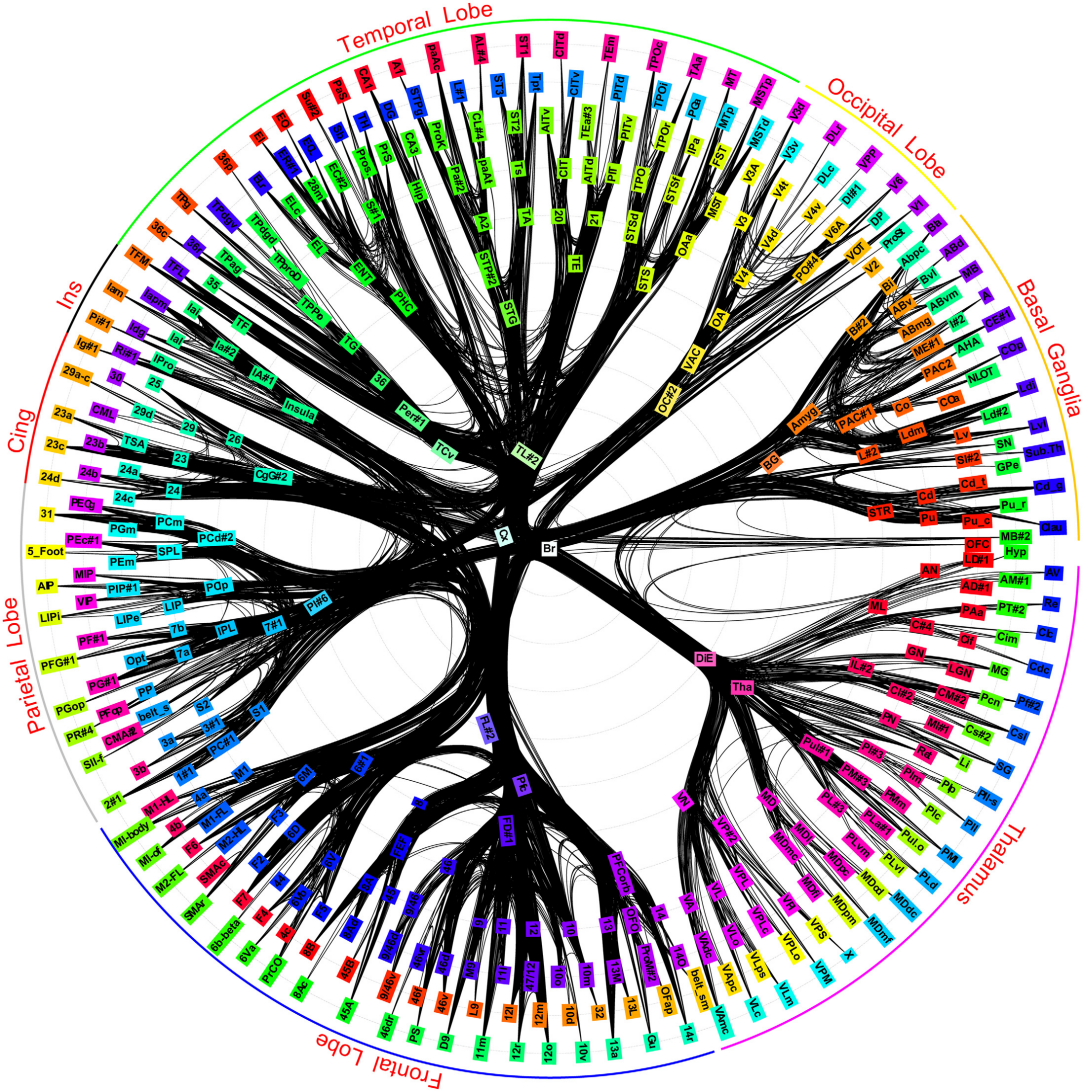
Macaque brain long distance network. Each vertex of the network corresponds to a brain region in the hierarchical brain map of Fig S6 of [2] and each edge encodes the presence of long distance connection between corresponding brain regions. A colorwheel is used for better discrimination amongst brain regions (vertices). For the leaf brain regions in the two outermost circles, the colorwheel is rotated by 120 and 240 degrees. Edges are drawn in black using algorithmically bundled splines.

The physical pathways can be modeled as a network where each brain area is a vertex and the presence of a reported long-distance connection is a directed unweighted edge between the corresponding vertices, as shown in Fig 1. There has been tremendous interest in topological analysis of these pathways; previous studies have provided a number of remarkable insights into the functioning of the brain including distributed and hierarchical structure of cortex [3], topological organization of the cortex [4], functional small-world characteristics, optimal set analysis, and multidimensional scaling [5], small-world characteristics [6], nonoptimal component placement for wire-length [7], structural and functional motifs [8], hub identification and classification [9], exponential degree distribution, tightly integrated core subnetwork [2], and rich club structure [10].

Much of the previous work has studied the densest subnetwork(s) of the brain network, for example the *k*-core [2], and the communities [11]. Dense subnetworks optimize path length at the cost of adding edges. In networks such as the brain’s, these subnetworks have functional implications—areas that are performing related functions tend to communicate more often, and hence increase their efficiency by having more connections [12]. For example, the *k*-core of the brain network is hypothesized to contain areas that belong to task-positive and task-negative functional networks [2, 13]. Thus studies of these dense subnetworks provide clues to the functionality of the brain. Note that in these studies the density of a subnetwork is relative to that of the network, hence it is a global property of the network.

A natural counterpoint to this theme that has not been explored as much is the study of sparse and local subnetworks. Sparse, connected, undirected networks of *N* vertices have order *N* edges; they optimize the number of edges at the cost of path length. Local subnetwork of a vertex, “hub”, is the network induced by its neighbouring vertices, “spokes” and itself. These hub-and-spokes networks are “stars” if they are absolutely sparse (*N* − 1 edges, and diameter of 2 [14]), or “cliques” if they are absolutely dense (*N* * (*N* − 1)/2 edges, and diameter of 1 [15]). In a star, the hub plays a central role in enabling shortest path communication between the spokes. As the network becomes denser by adding intra-spokes connectivity, the role of the hub decreases, such that in a clique the hub has no role in enabling shortest path communication.

In this paper we are interested in the role of a vertex in enabling communication between non-adjacent neighbouring vertices in an unweighted network, i.e., at most 2-hop connectivity. Though longer paths exist in the brain [2], they do not seem, either empirically or intuitively, to play as important a role in purposive neural interaction [16]. While many global communication models for the cortex, e.g., [17–19] have been proposed, in this work we assume that in a local neighbourhood, non-adjacent spokes communicate through the hub. Also there may be multiple hubs for each pair of spokes, but in our local, hub-centric viewpoint, these hubs are not aware of each other unless they are neighbours. To characterise the communication role of a vertex we introduce a novel metric, “star value” (SV), that measures how close its local subnetwork is to a star motif. We show that in the neural pathways network of Macaque and C.elegans there exists wide variation in roles—from hubs that are communicating with a large number of spokes with very few connections among them, to hubs that are communicating with a small number of interconnected spokes. In contrast, networks such as the Internet, power-grid, and realizations of Erdos-Renyi, Barabasi-Albert models have a very different distribution of communication roles.

This topological definition of communication role of a vertex can be further enriched if attributes are available for vertices of the network. In our dataset we have six cortical—Temporal Lobe, Occipital Lobe, Parietal Lobe, Frontal Lobe, Insula, Cingulate Gyrus—and two non-cortical—Basal Ganglia, Diencephalon—super-areas, and the brain areas are organised in a hierarchy such that each brain area belongs to a super-area. Each super-area is physically contiguous, distinct and functionally almost distinct from other super-areas; it encapsulates a notion of topography of the brain. Using its super-area membership as an attribute, we can further characterize the communication role of an area. We investigate the difference between the attribute values of a hub and its spokes and show that, as expected, most large star-like subnetworks exist across super-areas, and small cliques exist largely within a super-area. However, there are also star-like subnetworks within a super-area, implying that the brain conserves even short-distance (intra super-area) pathways.

Focusing on the case where the hub and spokes belong to a super-area, we ask are the super-areas “closed” for local communication? In other words, for a super-area are *all* pairs of non-adjacent spokes being connected by a hub in the super-area? Closure under local communication would be a desirable property for a super-area. Intriguingly we find that, in the brain no super-area is closed for communication and the degree of closure is different for different super-areas. For example, though the cingulate gyrus and the temporal lobe have approximately the same proportion of non-adjacent spokes, the temporal lobe contains far more hubs than the cingulate gyrus; thus the temporal lobe has a higher degree of closure than the cingulate gyrus. Note that by our convention vertices are spokes if they are connected to each other through a common hub, and unless otherwise stated we are interested in non-adjacent spokes.

In the substantial social science literature an interesting notion of “brokerage” has been proposed by Gould and Fernandez [20] where a vertex connected to two non-adjacent vertices brokers a transaction between these vertices. Five types of brokerage are defined using attributes of the vertices. We use this concept to create a brokerage profile for each vertex of the Macaque network, enumerating the number of times a vertex plays a brokerage type. We visualise the profiles using pie-charts and radial map of [2], and show, among other results, that not only the thalamic areas but also the insula and the cingulate gyrus areas often acts as mediators, or relays, in the Macaque brain. On the other hand, areas in the pre-frontal cortex and the hippocampus are mostly coordinating information within their respective super-areas. Note that our attributes are based only on the topological information and attribute derived from parcellation into super-areas. Detailed topographical information such as location, and functional implications may result in a more nuanced picture.

A recent paper [10, 21] studies “rich club” structure among areas with high degree of connectivity. Using network motif analysis they have shown that the rich club areas form star-like configurations. Though we agree with Harriger et.al.’s hypothesis that these configurations represent maximally centralized communication through a star’s hub vertex, objective of our study, methodology and results are completely different from theirs. In our study a star value is associated with each vertex due to its local subnetwork, not due to the aggregation of motifs. Topological properties of network have been used to define roles, for instance in [22], but our work is different because it is using the concept of brokerage [20] to further study communication role of a hub vertex. Also note that here hub denotes a vertex, unlike [9] where it denotes a high degree vertex.

## Results and Discussion

**Fig 2:**
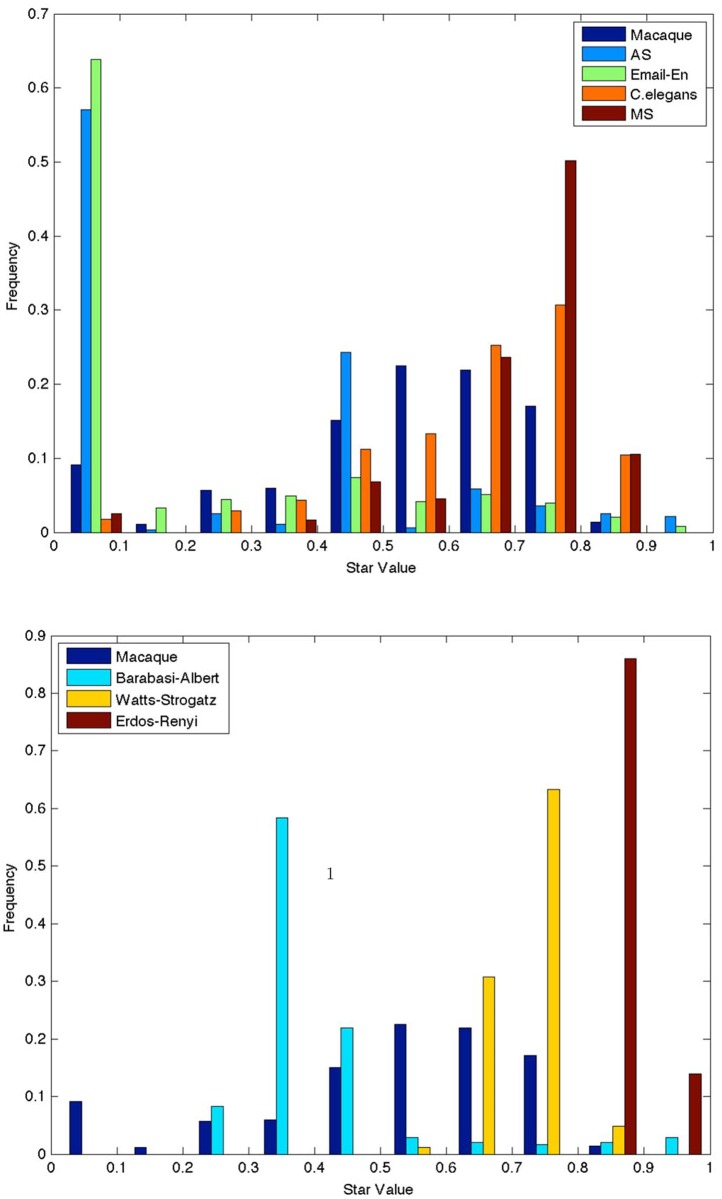
Normalized histogram of star value for undirected (a) Top plot: Real world networks, (b) Bottom plot: Random graph models along with Macaque network. MS is the null model of the undirected Macaque network. For random graph and null models results are averaged over 20 realizations (for fixed histogram bins, counts are average over realisations). The leftmost bin SV ≈ 0 counts clique-like motifs, while the rightmost bin SV ≈ 1 counts star-like motifs.

We begin by plotting normalized frequency distribution of SV, defined in Eq (2), of undirected real world networks (see Fig 2(a)) and random graph models (see Fig 2(b)). In an email network, there is minimal cost of adding an edge—people can often directly communicate with each other explaining the unimodal distribution with high frequency of occurrence of cliques in this network. In the Internet’s autonomous systems (AS) routing network, AS are organized in a hierarchy based on service profiles, geographical constraints and commercial agreements [23]. It can be argued that an AS network is constructed such that certain local subnetworks lower in the hierarchy are “replicated” so as to provide similar services. Thus motifs such as cliques, open triads, quads with an open triad occur often, explaining the bimodal distribution where local subnetworks with SV ≈ 0, and SV ≈ 0.5 occur with high frequency while other subnetworks occur with significantly lesser frequency.

Fig 2(b) shows that random graph models have heavily skewed SV distributions. Erdos-Renyi (ER) graphs have a preponderance of star-like motifs, which is to be expected given its low clustering coefficient (Table 1). Watts-Strogatz (WS) graphs constructed by rewiring a lattice have a larger clustering coefficient and thus result in local subnetworks with SV mainly in the mid to high range. On the other hand Barabasi-Albert (BA) graphs have local subnetworks with typically low to mid SV and some star-like subnetworks. The remarkable observation is that each of the random graph models has a preference for local subnetworks indicated by clear peaks within a small range of SV, i.e., high frequency of occurrence of subnetworks in a limited range of SV, and a significantly lower frequency for other subnetworks.

**Table 1:** Topological properties of the networks. Results for random graph models are averaged over 20 realizations. For the directed (undirected) network the largest strong (weak) connected component is used.

Network		No. of vertices	No. of Edges	Diameter	Characteristic Path Length	Average Clustering Coefficient	Average Star Value
Macaque	Directed	351	6602	6	2.62	0.330	0.386
Macaque	Undirected	351	10194	5	2.25	0.423	0.519
Celegans	Undirected	277	3836	6	2.62	0.277	0.633
AS	Undirected	6474	25144	9	3.70	0.252	0.240
Email-En	Undirected	33696	361622	11	4.02	0.491	0.181
Citation	Directed	7464	116252	35	9.01	0.184	0.117
Email-Eu	Directed	34203	151132	10	3.94	0.229	0.074
Watts-Strogatz	Undirected	351	10194	3	2.03	0.150	0.723
Barabasi-Albert	Undirected	351	10194	2	1.91	0.559	0.418
Erdos-Renyi	Undirected	351	10194	3	2.01	0.083	0.884

**Fig 3:**
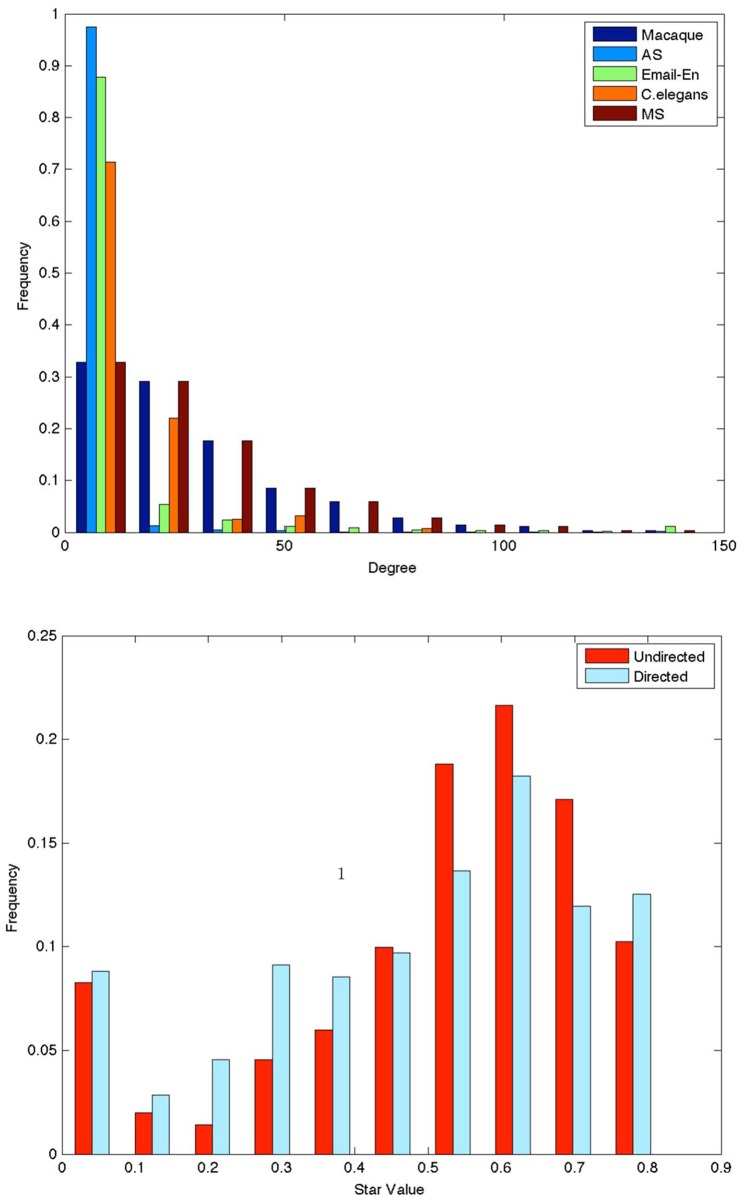
(a) Top plot: Normalized histogram of degree for undirected networks. (b) Bottom plot: Normalized histogram of star value for directed and undirected Macaque networks. We have not plotted the results for Citation or Email-EU because they are similar to undirected real-world networks’ results and we wanted to highlight the comparison between directed and undirected networks.

In contrast neural pathway networks have local subnetworks in a wide range of SV. Their frequency peaks at SV ≈ 0.7, but their frequency at other values of SV is not insignificant, especially in comparison with AS, and random graph models. Moreover, the distribution for the MS null model (Materials and Method) of the undirected Macaque network has a substantially different frequency distribution compared to that of the Macaque network with a higher peak at SV ≈ 0.8 and less clique-like motifs, though again it has *no* star-like motifs. The statistical measures, kurtosis and skewness, of the histograms in Fig 2 reported in Table 2 support our claim that the Macaque network’s star value distribution is different in that it is only-slightly right skewed and relatively flat as compared to that of the real world networks and random graph models. We have also plotted the degree distribution for the real world networks in Fig 3(a) for completeness sake.

**Table 2:** Skewness and Kurtosis of histogram, Fig 2. Note that same histogram bins are being used for real-world and random networks.

	AS	Email-En	Macaque	C.elegans	MS	Barabasi-Albert	Watts-Strogatz	Erdos-Renyi
Skewness	2.457	3.110	0.368	1.07	2.582	2.543	2.293	3.020
Kurtosis	6.038	9.762	-1.514	0.142	7.009	6.647	4.953	9.270

Fig 3(b) compares the SV distribution for directed and undirected Macaque networks using Eq (4) for directed SV. As expected from the definition of directed SV (see Methods and Materials for a discussion) there are a substantially higher number of clique like subnetworks, and slightly higher number of stars in the directed network. In Table 3 the ten hubs with the highest star value for the directed and the undirected Macaque network are listed. The hubs match well with the important vertices listed in [2] and occur mostly in the pre-frontal cortex and the parietal lobe. Interestingly for the directed network the area *SI*#2 “substantia innominata” in the basal ganglia is a hub. This area has the order of twenty sources and twenty targets areas, few of which are directionally connected. Hence its directed subnetwork is star-like. On the other hand if directionality is ignored then the number of connections between the adjacent areas goes up substantially, hence in the undirected case the subnetwork is *not* star-like; this points to the importance of directionality in network analysis [24]

**Table 3:** Top-10 hubs with star-like motifs.

Undirected	6#1	46	Insula	FD#1	13	PIT	IPL	32	PG#1	Opt
Directed	46	24	SI#2	13	PIT	7#1	Insula	6D	LIP	PS

Given the above results that show that local subnetworks of the Macaque brain have a distinctive distribution in the topology space, we next analyze how they are distributed in the attribute space. In this study attribute of an area is the super-area—lobes, diencephalon or basal ganglia—that it is a sub-area of, as identified by the hierarchical map of [2]. Thus the attribute codifies the notion of parcellation as captured by [2]. These results are on undirected Macaque network, unless otherwise stated.

**Fig 4:**
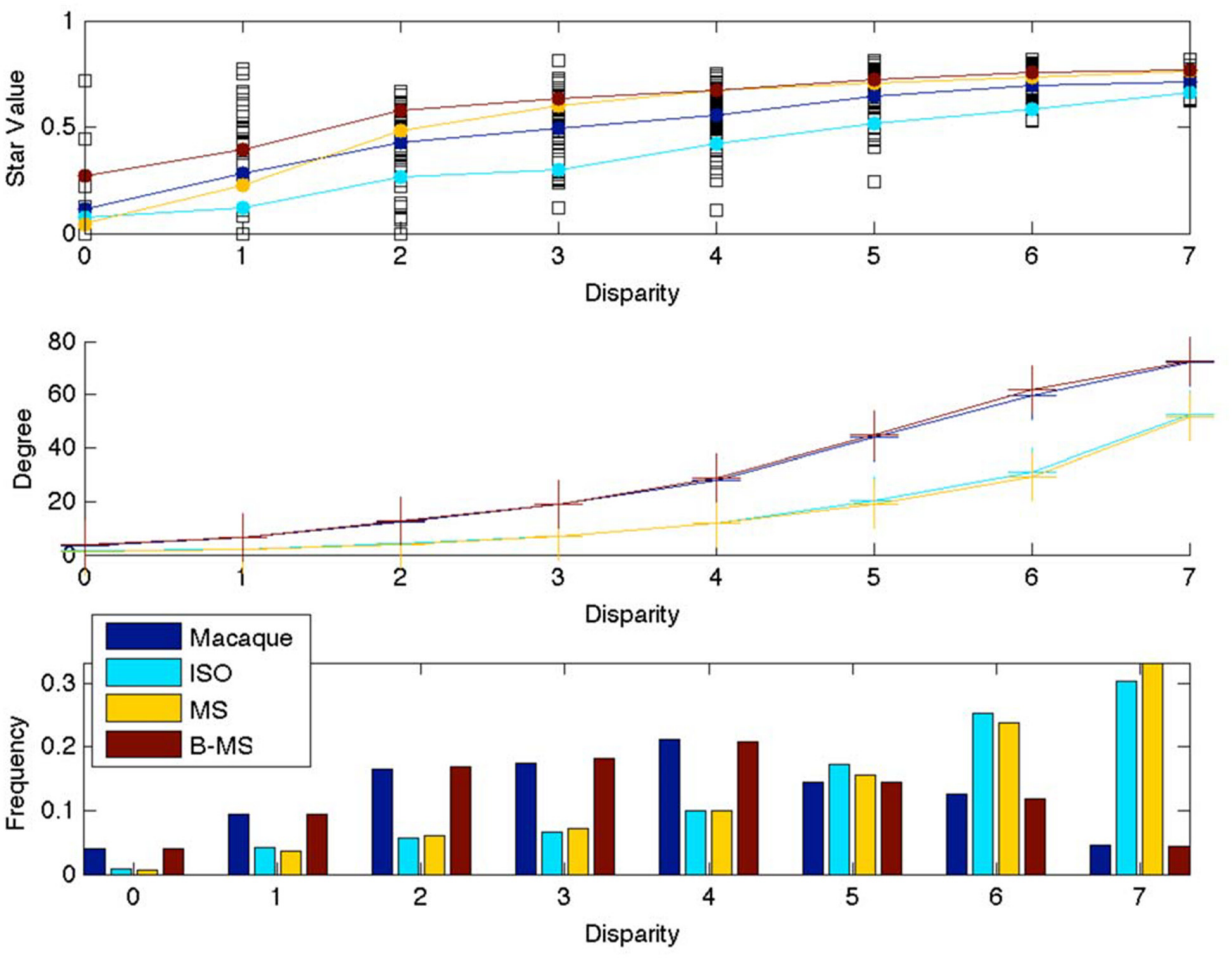
Disparity plots for undirected Macaque network and its null models. (a) Top plot: Black empty squares plot disparity against SV for all local subnetworks of the Macaque network. The mean SV of these subnetworks for each disparity value is plotted using the blue curve with filled circles. (b) Middle plot: the blue curve with crosses plots the mean degree of the hub of the subnetworks with the same disparity value. Mean values in the top and middle plot are plotted for different null models using the color legend shown in the bottom plot. (c) Bottom plot: Normalized histogram of local subnetworks for each disparity value, for each network. For ISO, MS, B-MS null models results are averaged over 20 realizations.

We first measure *disparity* (Methods and Material) in attribute space of each local subnetwork, and compare it against the disparity of null models of the network. As expected the *mean* SV is correlated with disparity, Fig 4(a). This implies that cliques occur largely within a super-area, while star-like subnetworks occur mostly across super-areas. However, note the overlap between the *actual* SV values (the squares) in the Macaque network at different disparity values; a star-like motif not only connects different super-areas, it also connects areas within a super-area, suggesting that even short edges (within a super-area) were added in the Macaque network if and only if needed—in the plot, even for low disparity, we expect (and we observe) more clique-like subnetworks, as wire length cost is minimal. But there are also star-like subnetworks, which is surprising because it implies that even relatively small distance pathways are added only if necessary. Fig 4(b) shows that the mean degree of hubs of local subnetworks is correlated with the disparity of the subnetworks. That is in the Macaque brain, on average, larger local subnetworks occur across super-areas. Fig 4(c) shows that the disparity histogram follows a bell shaped curve, that is the counts at mid disparity values are high, suggesting that most local subnetworks are connecting different super-areas. Comparing these results with those of null models, we can see that the Macaque network and its B-MS model have very similar results in Fig 4(b) and 4(c), while its ISO and MS model have substantially different results, suggesting that the intra super-area connections matter, further reinforcing organized complexity [2]. Though the SV frequency distribution of the ISO model and the Macaque network are identical, the higher mean SV for the Macaque network in Fig 4(a) implies that the attribute space, in our case the parcellation into super-area, matters. The B-MS model, which further preserves the attribute space, also has a consistently higher mean SV than the Macaque network, i.e. Macaque has more clique-like motifs than B-MS for the same number of edges. This suggests functional integration—the Macaque brain is optimizing functional constraints as and when needed.

**Fig 5:**
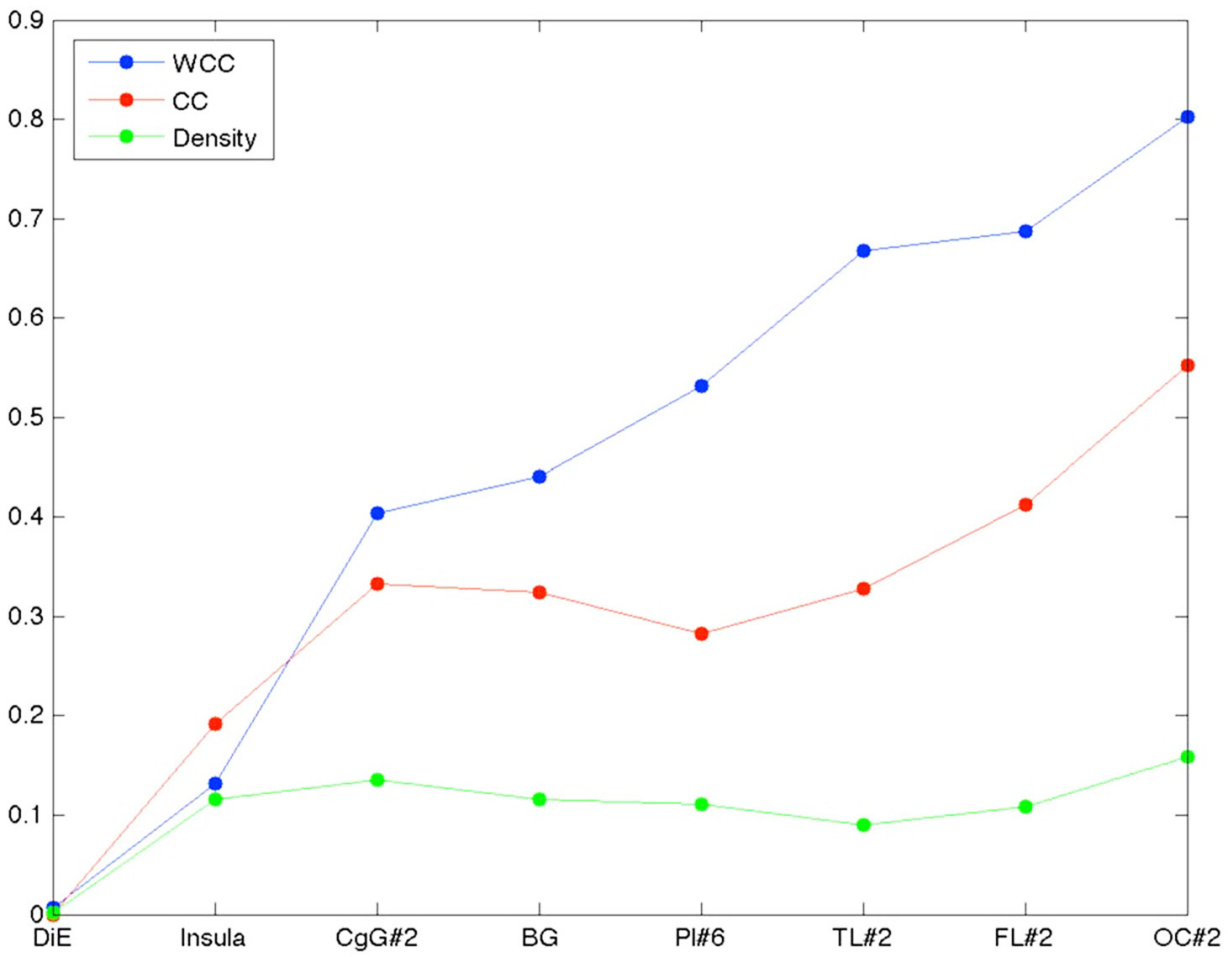
Workload Closure Coefficient plot. For each super-area “Density” is the number of connections within the super-area divided by the possible number of connections, “CC” is the clustering coefficient for the intra super-area subnetwork, and “WCC” is workload closure coefficient:- the number of closed pair of spokes divided by the total number of pair of spokes in a sub-area. The plot has been drawn such that WCC is sorted in ascending order, and the remaining two measures are sorted in the same order.

The above results show that very few local subnetworks are contained within a super-area, that is there is substantial communication between super-areas. But the converse question is of equal interest: can a super-area take care of all or most of its local communication? To answer this question we define a *workload closure coefficient* (WCC) (Materials and Method) for a super-area. Fig 5 shows that none of the super-areas can take care of their own communication workload by themselves. The occipital lobe has the highest WCC and it can take care of only about 80% of the available workload. In the figure we also plot the density and the clustering coefficient (CC) of each intra super-area subnetwork. While density influences the number of available pair of spokes, clustering coefficient is a measure of the number of spokes that directly communicate with each other; together these measures influence the available workload—the pairs of open triads. Note that, excepting the diencephalon, density is pretty uniform in the brain. Though CC does increase, it does not increase monotonically with WCC. Thus although the temporal lobe has more open triads than say the cingulate gyrus, the former takes care of a larger share of its workload. This suggests that connectivity is organized within the super-area in a manner that is not captured by traditional measures like CC and density. It should also be noted that neocortical areas have a higher WCC in general, and that insula, which has a lower WCC than CC, has few open triads that are being served mostly by areas outside insula.

We now look at Gould and Fernandez’s *brokerage* types (Materials and Method). A super-area would be workload closed if there were no “consultants” for that super-area. The above result shows that every super-area in the Macaque brain has a consultant area in another super-area. We now investigate this and other brokerage types in more detail. A hub in a network can be a different type of broker for different pairs of spokes. Counting the number of times a hub is a particular type of broker we draw a pie-chart for the hub, with the size of a slice of pie being proportional to the count of a type. We replace the vertex icons in the Macaque network visualization of Fig 1 (originally from [2]) with icons of these pie-charts.

**Fig 6:**
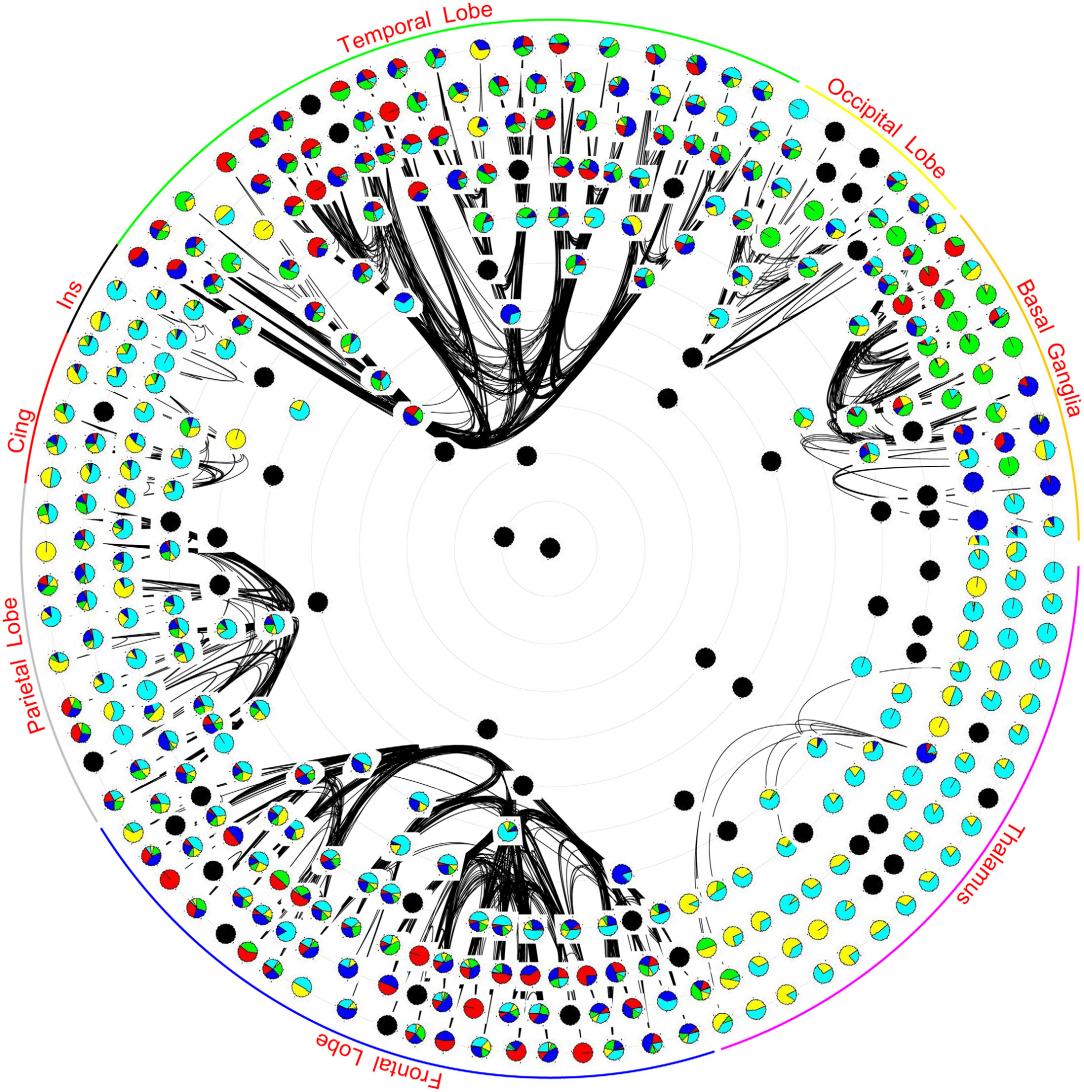
Vertices are laid out using the hierarchical map and radial layout of Fig 1. The pie-chart for each vertex is based on unnormalized counts of its brokerage type. In the pie-chart red color is for coordinator, blue for gatekeeper, green for representative, yellow for consultant and cyan for liaison. The black splines are the intra-lobe connectivity. The blackened vertices are those that have either no connectivity or have been assigned no roles by UCINET.

**Fig 7:**
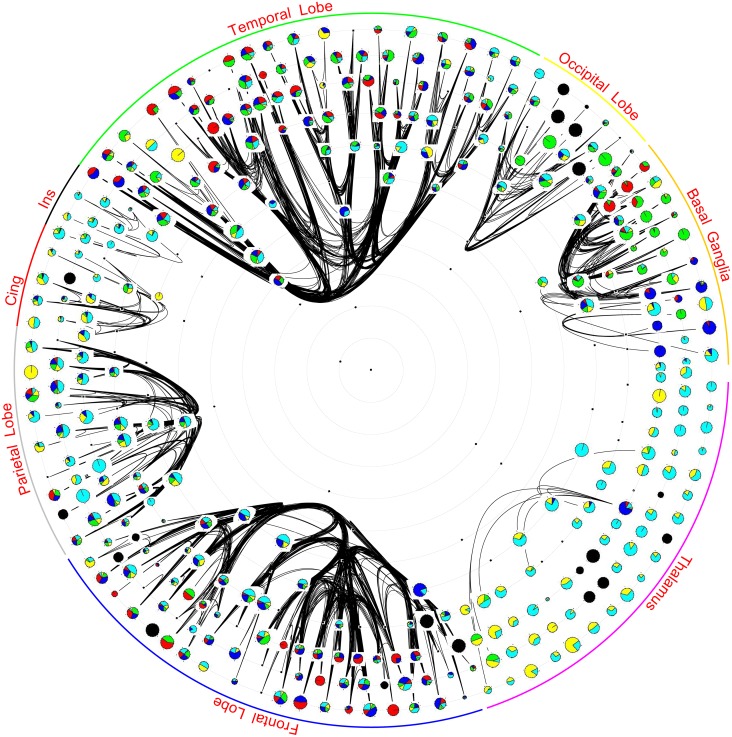
Vertices are laid out using the hierarchical map and radial layout of Fig 1. The pie-chart for each vertex is based on unnormalized counts of its brokerage type. In the pie-chart red color is for coordinator, blue for gatekeeper, green for representative, yellow for consultant and cyan for liaison. Size of each vertex is proportionate to its star value. The black splines are the intra-lobe connectivity. The blackened vertices are those that have either no connectivity or have been assigned no roles by UCINET.

In Fig 6, unnormalized raw counts are used for the pie-charts while in Fig 7 the size of icons is additionally rescaled using the star value of the vertex—larger star value implies larger icons. Most thalamic areas play the role of consultant or liaison due to the lack of intra thalamic connections. However note from Fig 5 that the density of all other intra super-area subnetworks is approximately the same. Hence the preponderance of consultant and liaison roles in insula and cingulate gyrus, and to a lesser extent in the parietal lobe, suggests that these super-areas often play a role in mediating, and transforming, information between other super-areas. In basal ganglia, which has a near clear segregation of representative, gatekeeper and coordinator roles, areas that are laid out closer to the occipital lobe often play the coordinator role, those closer to thalamus the gatekeeper role, and those in the middle play the representative role, Fig 6. Note that the layout of this figure has been ordered by using a concept of wire length minimisation—areas that are connected tended to lie close together in the figure [2].

In the occipital lobe, most areas seem to play all roles in varying degrees. The lack of coordinators, despite the large WCC, is because its CC is very high—there are not many open triads in the occipital lobe. Frontal and temporal lobes are the two super-areas that have more than a fair share of coordinators consistent with the observation that their WCC is reasonably high. While in the temporal lobe coordinators are mostly in the hippocampus, in the frontal lobe they are in the prefrontal cortex. The diversity of roles in the occipital lobe also lends support to the indeterminacy of a unique visual hierarchy [25] argument. Fig 7 shows that star value and brokerage types are not correlated and that there is no apparent preference for a star value in a super-area.

Table 4 lists the top-10 areas for each brokerage type, while Tables 5–9 list the top area for a particular brokerage type for each pair of super-area. These areas have the highest unnormalized counts for their particular role. A “–” indicates that either that the role is not possible or is not reported. The top-10 areas are consistent with the important areas listed in [2]. The representative areas in the occipital lobe are the ones higher in the visual hierarchy [3], and consultant, liaison brokerage for occipital lobe is performed by areas in the parietal lobe. Similar observations can be made regarding other super-areas. This work is the first complete enumeration of the brokerage type for areas in a primate brain that we are aware of.

**Table 4:** Top 10 areas for each brokerage role. Unnormalized brokerage values are used.

Coordinator	TF	AITv	46	32	TH	12o	ENT	PIT	AITd	TE
Representative	46	32	12o	F7	13	12l	TF	46v	9	13a
GateKeeper	46	TF	TE	TH	13a	PIT	TG	32	36	9
Consultant	24	46	MD	13	13a	24c	LIP	32	F7	M1
Liason	24	46	LIP	13	MD	32	PGm	13a	9	PM#3

**Table 5:** Best Coordinator Area.

	DiE	BG	OC#2	TL#2	Pl#6	FL#2	CgG#2	Insula
DiE	Ret	-	-	-	-	-	-	-
BG	-	L#2	-	-	-	-	-	-
OC#2	-	-	V2	-	-	-	-	-
TL#2	-	-	-	TF	-	-	-	-
Pl#6	-	-	-	-	7b	-	-	-
FL#2	-	-	-	-	-	46	-	-
CgG#2	-	-	-	-	-	-	23c	-
Insula	-	-	-	-	-	-	-	Ig#1

**Table 6:** Best Gatekeeper Area.

	DiE	BG	OC#2	TL#2	Pl#6	FL#2	CgG#2	Insula
DiE	-	L#2	V2	PIT	PGm	32	24	Iai
BG	Pul#1	-	V1	TF	S2	13	24	Ig#1
OC#2	Pul#1	Cd_g	-	TF	LIP	46	24c	-
TL#2	Ret	L#2	V2	-	7b	46	24	Idg
Pl#6	Ret	Cd_g	V2	TF	-	46	23c	Ig#1
FL#2	Ret	Ldi	V4	ENT	S2	-	24	Iai
CgG#2	Ret	Lv	-	ENT	7b	46	-	Iai
Insula	Ret	Lv	-	TH	S2	46	24	-

**Table 7:** Best Representative Area.

	DiE	BG	OC#2	TL#2	Pl#6	FL#2	CgG#2	Insula
DiE	-	-	LGN	MD	MD	MD	MD	MD
BG	Abpc	-	Bla	L#2	ABmg	Abpc	L#2	L#2
OC#2	V4	V2	-	V4	V6	PO#4	V3	-
TL#2	TG	TF	TF	-	TF	TE	TH	TE
Pl#6	PECg	PECg	LIP	LIP	-	7b	7b	7b
FL#2	46	13a	46	46	46	-	46	13a
CgG#2	24	24	-	24	23c	24	-	24
Insula	Ia#2	Iai	-	Iai	Idg	Iai	Iai	-

**Table 8:** Best Consultant Area.

	DiE	BG	OC#2	TL#2	Pl#6	FL#2	CgG#2	Insula
DiE	24	-	-	-	-	-	-	-
BG	-	36r	-	-	-	-	-	-
OC#2	-	-	LIP	-	-	-	-	-
TL#2	-	-	-	46	-	-	-	-
Pl#6	-	-	-	-	VPL	-	-	-
FL#2	-	-	-	-	-	MD	-	-
CgG#2	-	-	-	-	-	-	46	-
Insula	-	-	-	-	-	-	-	SI#2

**Table 9:** Best Liason Area.

	DiE	BG	OC#2	TL#2	Pl#6	FL#2	CgG#2	Insula
DiE	-	32	LIP	46	M1	24	46	24
BG	13	-	TF	13	24	MD	13	MD
OC#2	MT	PIT	-	LIP	VPL	LIP	8A	LIP
TL#2	24	13a	LIP	-	46	MD	46	13a
Pl#6	M1-FL	46	PIT	46	-	23c	46	F5
FL#2	24	24	LIP	24	24	-	36	24
CgG#2	46	46	46	46	46	MD	-	7b
Insula	13	36	LIP	46	46	24	46	-

**Fig 8:**
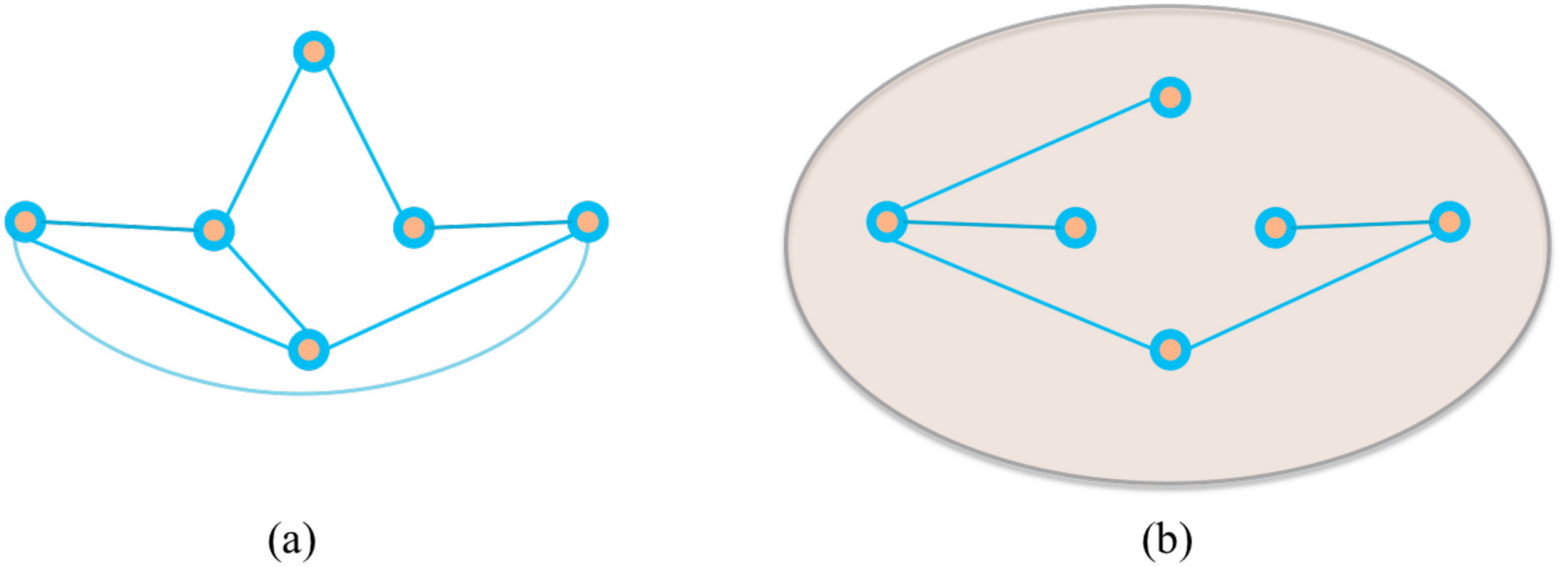
In (a) there are very few open triads generating a low workload among the vertices. Regardless of whether the spokes and their hubs have the same attribute or not, the workload closure (WCC) will be high because of the low workload. In (b) there are more open triads, but all are being served by hubs having the same attribute as their spokes. Hence though the workload is high, the WCC is also high. Note that in this paper attribute, is the membership of a super-area.

In conclusion, this paper explores local neighbourhoods of vertices in the Macaque brain network and by measuring how sparse they are, it categorizes the neighbourhoods in terms of workload on the hub. We have shown that the local neighbourhoods in the Macaque brain network are fairly diverse compared to both other real world networks (including those we have studied but not reported here), and random graph models. This result raises questions about how the brain network evolved? Clearly not through the replication model of AS, nor the random model of ER, and likely not even due to the scale-free evolution of BA graphs. Our findings support recently proposed models for brain functional network that tradeoff competing factors [26]. There are no stars in the Macaque brain or in its MS null model. However, the frequency distribution is right skewed indicating a preference for higher star values. All of this implies that the brain is not just optimizing for wire-length [27], it is also optimizing for hop delay and functionality [7]. This is further reinforced by Fig 4 which shows that though star-like structures exist across super-areas, typically across 4 super-areas, they also occur within a super-area. Shifting focus from local neighbourhoods to pairs of non-adjacent spokes, in order to study communication role in more depth, we introduced the measure of workload closure; we can intuitively argue that there is a relationship between density, CC and WCC. Density counts the number of spokes, CC the number of closed triads, the WCC is a function of the number of open triads served within the super-area, see Fig 8 for an example. The plot of Fig 5 suggests that the relationship is determined by the arrangement of edges in the super-area and needs further investigation. A super-area is considered to be workload closed if it has no consultants such as the occipital lobe where WCC is high, which is not surprising given that CC is high—there are very few open triads to generate workload. In general, study of brokerage roles of brain areas in the long distance pathway concepts is novel, and though it can be argued that a brain area does more than just brokerage, our analysis enumerates the nature of brokerage interactions in the brain. As stated earlier this analysis is predicated on both the network data and the attribute data, the hierarchical divisions into super-areas and further into areas. Also its possible to generate topological hierarchical divisions of the network using community detection algorithms, e.g., [28], but in this work we are interested in the topographical information as codified by the mapping data in CoCoMac. As higher resolution mapping data becomes available a more nuanced analysis of communication roles can be done.

## Materials and Methods

### Data Sets

We study the long range network of the Macaque brain as derived by Modha and Singh [2]. The network is based on anatomical tracing studies of the Macaque brain compiled by the online database CoCoMac [29]. Given the resolution of anatomical tracing experiments, the database typically furnishes data at a macroscale of cortical areas or, more generally, brain regions. It covers 383 cortical and sub-cortical brain areas and codes the presence of 6602 directed projections between these areas. The brain areas are arranged in a hierarchical brain map, which is consistent with a recursive parcellation of the brain [2]. 351 of the 383 areas have connectivity; the remaining areas are container or super-areas that hold the hierarchy together. We differentiate a super-area from a brain area in that a super-area is sub-divided into brain areas and it does not report any projections. The hierarchical map divides the brain (Br) into basal ganglia (BG), diencephalon (DiE), and cortex (Cx). Cortex is divided into 6 lobes, temporal (TL#2), occipital (OC#2), parietal (Pl#6), frontal (FL#2), cingulate gyrus (CgG#2) and insula (Ins). These super-areas are further sub-divided into other super-areas and brain areas. By ignoring the directionality of projections we also create an undirected network that has 10194 undirected edges. In this paper, we compare the Macaque network against the following real-world networks and random graph models. The basic statistics of the networks are shown in Table 1.

**AS** The graph of routers comprising the Internet can be organized into sub-graphs called Autonomous Systems (AS). Each AS exchanges traffic flows with some neighbours (peers). We can construct a communication network of who-talks-to-whom from the BGP (Border Gateway Protocol) logs. The data is an instance of Autonomous Systems graph from January 02, 2000 from University of Oregon Route Views Project—Online data and reports [30]. A vertex represents an AS and an undirected edge between vertex *i* and vertex *j* represents exchange of traffic between the two corresponding AS.
**Email-En** Enron email communication network covers all the email communication in a dataset of around half million emails. This data was originally made public and posted to the web by the Federal Energy Regulatory Commission during its investigation. A vertex represents an email address and an undirected edge between vertex *i* and *j* represents exchange of atleast one email between the two corresponding addresses [31, 32].
**Email-EU** A large European research institution email communication network covers the email communication in a period of 18 months starting October 2003 [30]. A vertex represents an email address and a directed edge between vertex *i* and *j* represents exchange of at least one email message from *i* to *j*.
**Citation** This is the Arxiv HEP-TH (high energy physics theory) citation network from [30]. A vertex represents a paper and a directed edge from vertex *i* to vertex *j* represents that paper *i* has cited paper *j*. The data covers papers in the period from January 1993 to April 2003 (124 months).
**C.elegans** The neural network of *Caenorhabditis elegans* [33]. This dataset has 277 neurons and each edge represents a directed synaptic projection between two neurons [7]. We are using the undirected version of this network.
**Erdos-Renyi** ER [34] model generates undirected random graphs of *n* vertices with each edge occurring independently with probability *p*. This is realized using the igraph package [35].
**Barabasi-Albert** BA [36] model generates undirected random graphs using a preferential attachment mechanism such that their degree distribution of the vertices asymptotically follow a power law. This is realized using the igraph package [35].
**Watts-Strogatz** WS [37] model generates undirected graphs with small world properties, that is graphs that have short average path lengths, and high clustering coefficient. This is realized using the igraph package [35].

### Null Models

Null models used in this work are: (i) networks that are isomorphic to the original network, (ISO), (ii) randomized networks that preserve the degree of each vertex by using Maslov and Sneppen switching algorithm [38] (MS), and (iii) randomized networks that preserve the degree of each vertex, and the sparsity of the inter and intra super-area subnetworks (B-MS).

The degree distribution of the ISO network is the same as that of the original network but the degree sequence and attribute values are changed because the vertices are permuted. On the other hand in the MS model the degree distribution, degree sequence, and the attribute value of vertices are maintained, but the number of edges in the inter and intra super-area subnetworks changes. B-MS model extends this model by using Maslov and Sneppen algorithm on each inter, and intra super-area subnetwork independently, such that the number of edges in these subnetworks are maintained along with the degree sequence. Hence B-MS model is most similar to the original network in that it not only preserves the attribute space and the degree sequence but also preserves the sparsity of edges in the inter and intra super-area subnetworks.

### Star Value

An undirected star network of *n* vertices has *n* − 1 edges that connect a central vertex, the hub, to the remaining *n* − 1 vertices, the spokes. A subnetwork defined by a vertex (hub), and its adjacent vertices (spokes) in an undirected network is a star *iff* the adjacent vertices have no edges between them. Study of such subnetworks is important because the hub plays a central role in providing the shortest path connectivity between the spokes. A star subnetwork can be generalized by allowing connectivity between the spokes, and in turn reducing the role of the hub. As the spokes get more interconnected the role of the hub decreases, and finally in a clique the hub has no role in connecting the spokes. In this paper we propose a novel measure called “star value” (SV) which measures the role of a vertex in communication among its adjacent vertices.

Given an undirected network with no weights on its edges *G* = (*V*, *E*), let *v*_*k*_ be a vertex in *G* that has a set *C*_*k*_ of adjacent vertices, with *m*_*k*_ edges between the adjacent vertices. The communication workload on *v*_*k*_ is,

$$L\;\left(v_{k}\right)=\frac{∥C_{k}∥(∥C_{k}∥-1)}{2}-m_{k}.$$ (1)

where ‖ ⋅ ‖ represents the cardinality of a set. For ‖*C*_*k*_‖ spokes there are $\frac{∥C_{k}∥(∥C_{k}∥-1)}{2}$ pairs that are communicating through the hub. From this the contribution of the pairs that are communicating directly with each other, which is *m*_*k*_, is subtracted to arrive at the workload of the hub. The assumptions here are that communication is along the shortest hop paths and though there maybe multiple parallel short paths available, the hub-centric viewpoint is that the communication is happening due to the hub. The assumptions can be modified if the mode of communication such as broadcast, point-point, or any other additional information is available. The star value *SV*(*v*_*k*_) is the normalized workload such that for a clique it is zero and for a star it tends to one as ∣∣*C*_*k*_∣∣ grows,

$$SV\;\left(v_{k}\right)=\frac{2\;*\;L\;(v_{k})}{\left|\right|C_{k}{\left|\right|}^{2}}=1-\frac{2\;*\;m_{k}+∥C_{k}∥}{∥C_{k}{∥}^{2}}.$$ (2)

For an undirected network the star value is a measure of the density of a local network. It is related to clustering coefficient [37] and its variants local efficiency [39], and broker measure [40]. Below we list what we believe are the main differences between the clustering coefficient (CC) and SV.

- CC is useful in a social network analysis measuring the “friend of a friend” effect, but it ignores the “friends who are not friends of friends” effect. Due to the plurality of the latter effect, the size of local subnetwork matters.
- CC is a measure of the inter-spoke communication in a neighbourhood, while SV is a measure of the inter-spoke *and* the hub-spoke communication in a neighbourhood. Again the size of the neighbourhood matters.
- CC is zero for all stars. It does not distinguish between a small and a large star. Thus larger stars would mean that there are more friends that are not friends of each other and there is more communication through the hub.
- CC is one and SV is zero for all cliques. Here size does not matter because the hub is no different from any of its spokes in terms of connectivity.

**Fig 9:**
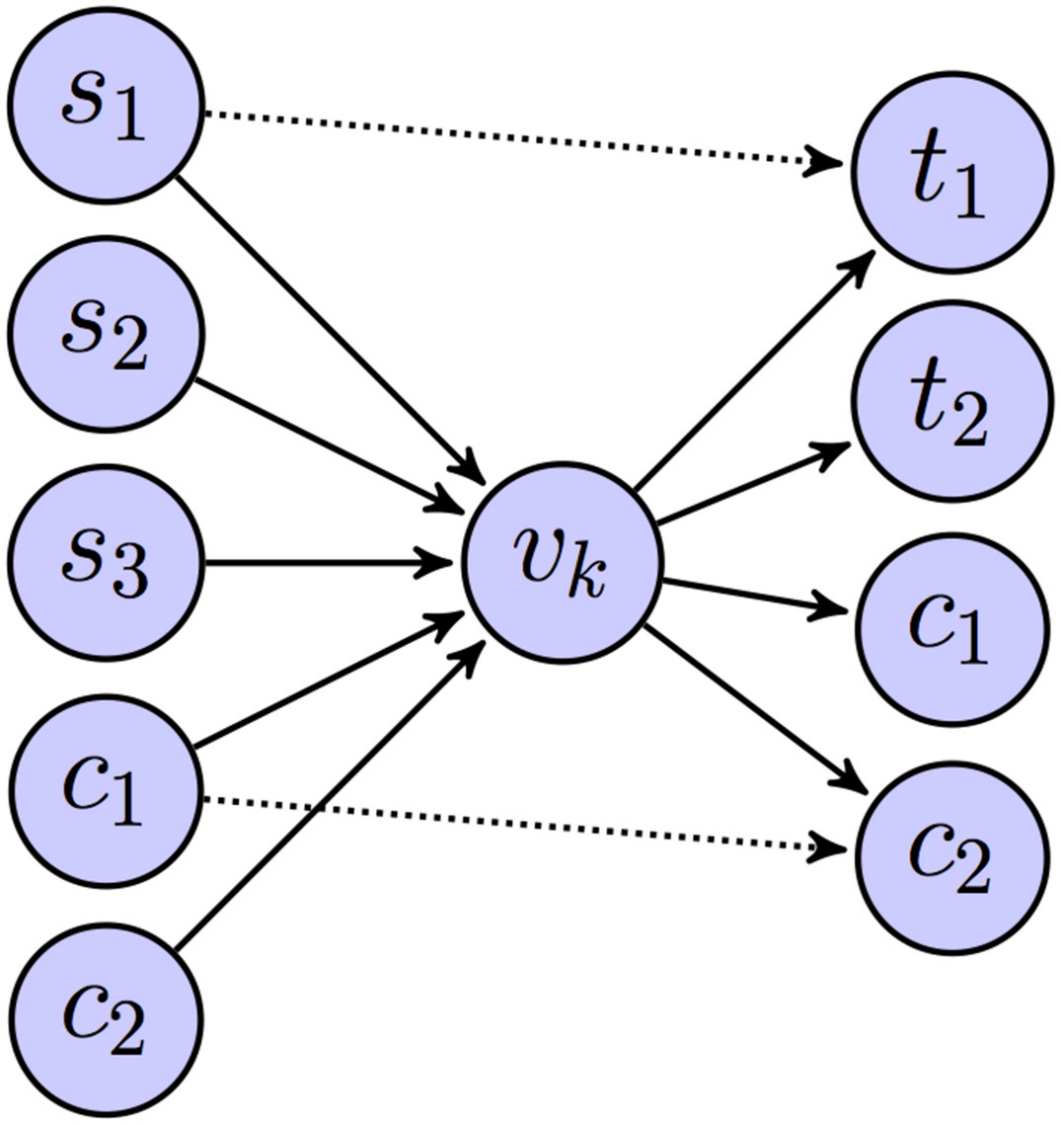
The local subnetwork of hub vertex *v*_*k*_ modeled as a tripartiate graph—*S*_*k*_ + *C*_*k*_ vertices in the first partition, *v*_*k*_ vertex in the second partition, and *T*_*k*_ + *C*_*k*_ vertices in the third partition. The intra-spokes edges are directed from the vertices in the first partition to the vertices in the third partition, shown by dotted arrows.

It is for directed graphs, however, that the concepts of SV and CC are fairly distinct. SV captures the communication load on a hub and hence actually enumerates the set of afferent and efferent connections on the hub—we are not aware of any other similar measure. In a directed network let there be a hub *v*_*k*_ with a set *S*_*k*_ of vertices that have directed edges *E*_*S*_*k*_,*v*_*k*__, a set *T*_*k*_ of vertices that have directed edges *E*_*v*_*k*_,*T*_*k*__, and a set *C*_*k*_ of vertices that have directed edges *E*_*C*_*k*_,*v*_*k*__ and *E*_*v*_*k*_,*C*_*k*__ as shown in Fig 9. Vertices in the source set ${\tilde{S}}_{k}=S_{k}\cup C_{k}$ are communicating with vertices in target set ${\tilde{T}}_{k}=T_{k}\cup C_{k}$ through the hub *v*_*k*_. Also let the number of directed edges from source set to target set be *m*_*k*_. Then the communication workload for *v*_*k*_ is,

$$L\;\left(v_{k}\right)=∥S_{k}∥\;*\;(∥T_{k}∥+∥C_{k}∥)+∥C_{k}∥\;*\;(∥T_{k}∥+\frac{∥C_{k}∥-1}{2})-m_{k}$$ (3)

Star value is the normalized workload such that it is between 0 and 1,

$$SV\;\left(v_{k}\right)=\frac{L\;(v_{k})}{(∥S_{k}\;*\;(∥T_{k}∥+∥C_{k}{∥+2)∥}^{2}+∥T_{k}∥\;*\;(∥C_{k}∥+1)+\frac{∥C_{k}{∥}^{2}}{2})}$$ (4)

It is easy to see that this definition is consistent with the undirected case given above; in an undirected network *T*_*k*_ = *S*_*k*_ = ∅, and *C*_*k*_ represents the set of adjacent vertices. The subnetwork in Fig 9 is a “clique” if all vertices in the first partition are directionally connected to all vertices in the third partition; intra partition vertices need not be connected. Hence directed cliques could have upto one fourth the number of edges in an undirected clique with the same number of vertices. On the other hand directed stars can be denser than undirected stars; the only requirement is that there should be no directed connections from vertices in the first partition to vertices in the third partition; other edges if they exist are not counted.

### Attribute

For the purpose of attaching an attribute to a brain area we identify a set of 8 super-areas: BG, DiE, TL#2, OC#2, Pl#6, FL#2, CgG#2 and Ins. For each brain area we identify from this set the super-area it belongs to, and assign a unique integer between one and eight as an attribute value to the corresponding vertex. For example all areas in BG are assigned attribute value 1, those in TL#2 are assigned attribute value 3, and so on.

### Disparity

Disparity for a local subnetwork is a measure of the heterogeneity of the hub and spokes in terms of their attribute values. It is defined as the cardinality of the set difference of the attribute values of the spokes and the attribute value of hub. A disparity of zero implies homogeneity as the hub and spokes all belong to the same super-area. If attribute values are in the range [1…*K*], the maximum disparity is *K* − 1 and implies that the subnetwork has vertices from all the different super-areas.

### Workload Closure Coefficient

A pair of spokes are “workload closed” if they and atleast one of their hubs have identical attribute values. A group of vertices having identical attribute values is workload closed if for all pairs of spokes in the group, one of their hubs is also in the group. A closed group has the interesting property that all intra-group communication can be served by vertices in the group. We define workload closure coefficient (WCC) of a group as the proportion of number of pairs of spoke in the group that are closed, to the total number of pairs of spokes in the group. All the pair of spokes in a group, and the subset pair of spokes that have a hub in the group are enumerated to calculate the coefficient. Note that for this paper group is the set of areas in a super-area.

### Brokerage

To define brokerage for directed networks, lets use the convention that the source vertex *s* is directionally connected to the broker *b*, who is directionally connected to the target vertex *t*. Let *A*(*v*) denote the attribute value of a vertex *v*. Here we are assuming real-valued attributes. All vertices having the same attribute belong to the same group. The five types of brokerage are named using terminology from social roles [20].

**Coordinator** *b* is a broker such that *A*(*s*) = *A*(*b*) = *A*(*t*), that is all three vertices belong to the same group.
**Gatekeeper** *b* is a broker and *A*(*s*) ≠ *A*(*b*) and *A*(*b*) = *A*(*t*), that is the source vertex belongs to a different group.
**Representative** *b* is a broker and *A*(*s*) = *A*(*b*) and *A*(*t*) ≠ *A*(*b*), that is the destination vertex belongs to a different group.
**Consultant** *b* is a broker such that *A*(*s*) = *A*(*t*), but *A*(*b*) ≠ *A*(*s*), that is the broker belongs to one group, and the source and target vertices belong to another group.
**Liaison** *b* is a broker and *A*(*a*) ≠ *A*(*b*) ≠ *A*(*c*), that is each vertex belongs to a different group.

We used UCINET [40] for finding the brokerage types for each vertex. We selected the “unweighted” option for our analysis. The unnormalized brokerage value simply counts the number of times a given vertex is in a brokering position, regardless of how many other vertices are serving the same function with the same pair of spokes. On the other hand a relative brokerage value is the unnormalized value divided by the expected value of each brokerage measure given the number of groups and the size of each group. The expected value is based on the assumption that the network can be modeled as an Erdos-Renyi graph. As this is not true for the Macaque network, we use only the unnormarlized counts in the current work. Note that in this work for the discussion of brokerage, role is used interchangeably with brokerage type.

### Kurtosis and Skewness

In probability theory and statistics, kurtosis is a measure of the “peakedness” of the probability distribution of a real-valued random variable, while skewness is a measure of the asymmetry of the probability distribution of a real-valued random variable. Positive kurtosis indicates a relatively peaked distribution. Negative kurtosis indicates a relatively flat distribution. Positive skewness indicates a distribution with an asymmetric tail extending toward more positive values. Negative skewness indicates a distribution with an asymmetric tail extending toward more negative values. The definitions we are using here are described in [41].
